# Widespread Structural and Functional Connectivity Changes in Amyotrophic Lateral Sclerosis: Insights from Advanced Neuroimaging Research

**DOI:** 10.1155/2012/473538

**Published:** 2012-06-10

**Authors:** Francesca Trojsi, Maria Rosaria Monsurrò, Fabrizio Esposito, Gioacchino Tedeschi

**Affiliations:** ^1^Department of Neurological Sciences, Second University of Naples, Piazza Miraglia 2, 80138 Naples, Italy; ^2^Neurological Institute for Diagnosis and Care “Hermitage Capodimonte”, Via Cupa delle Tozzole 2, 80131 Naples, Italy; ^3^Magnetic Resonance Imaging Center, Italian Foundation for Multiple Sclerosis (FISM), Via Cupa delle Tozzole 2, 80131 Naples, Italy; ^4^Department of Neuroscience, University of Naples Federico II, Via S. Pansini 5, 80131 Naples, Italy

## Abstract

Amyotrophic lateral sclerosis (ALS) is a severe neurodegenerative disease principally affecting motor neurons. Besides motor symptoms, a subset of patients develop cognitive disturbances or even frontotemporal dementia (FTD), indicating that ALS may also involve extramotor brain regions. Both neuropathological and neuroimaging findings have provided further insight on the widespread effect of the neurodegeneration on brain connectivity and the underlying neurobiology of motor neurons degeneration. However, associated effects on motor and extramotor brain networks are largely unknown. Particularly, neuropathological findings suggest that ALS not only affects the frontotemporal network but rather is part of a wide clinicopathological spectrum of brain disorders known as TAR-DNA binding protein 43 (TDP-43) proteinopathies. This paper reviews the current state of knowledge concerning the neuropsychological and neuropathological sequelae of TDP-43 proteinopathies, with special focus on the neuroimaging findings associated with cognitive change in ALS.

## 1. Introduction

Amyotrophic lateral sclerosis (ALS), also known as motor neuron disease, is a progressive disorder causing degeneration of the motor system at all levels, from the cortex to the anterior horn of the spinal cord. Approximately 5% of cases are familial, whereas the bulk of patients diagnosed with the disease are classified as sporadic as they appear to occur randomly throughout the population. A large hexanucleotide repeat expansion in the first intron of the C9ORF72 gene is resulted, the most common genetic cause of familial ALS (FALS). It was detected in more than one-third of FALS cases of European ancestry and in nearly one-half of Finnish FALS cases [1].

Despite the early view of ALS as a neurodegenerative disease that exclusively affects the motor system, growing evidence supports the new concept of ALS as a multisystem disease also affecting executive functions, behavior, language, and other cognitive domains, functionally associated, in general, with temporal and frontal lobes [2, 3]. A detectable, although variable in magnitude, degree of cognitive involvement has been found in many patients with ALS. Indeed, 5–15% of ALS patients meet criteria for frontotemporal dementia (FTD), while a substantial percentage of patients without dementia may show mild to moderate executive (approximately from 22 to 35%) and behavioral (up to 63%) dysfunctions [3–5]. In support of these clinical evidences, immunohistochemical findings suggest that ALS may affect the frontotemporal network and, furthermore, is considered part of a broader clinicopathological spectrum now known as TAR-DNA binding protein 43 (TDP-43) proteinopathies which also include FTD [6, 7].

Structural and functional magnetic resonance imaging (MRI), positron emission tomography (PET), and single photon emission-computed tomography (SPECT) studies have corroborated the theory of frontotemporal impairment in ALS with approximately half of the patients displaying at least mild abnormalities [8–25]. In particular, the diffusion tensor imaging (DTI) findings of reduced white matter (WM) integrity in the frontal, temporal, and parietal lobes and in the corpus callosum suggest that a widespread WM involvement may underlie both cognitive and functional changes in ALS [17–25].

As the brain is a complex system of interacting structures, relevant contributions have been derived from resting-state functional magnetic resonance imaging (RS-fMRI), a novel technique that evaluates the spontaneous fluctuations in the Blood Oxygen Level-Dependent (BOLD) signals with subjects being completely at rest [26, 27], which proved to be particularly suitable to explore functional interactions between cerebral networks in ALS. Indeed, local degeneration of motor neurons was found to be accompanied by a widespread effect on brain networks [21, 22, 28, 29]. A whole body of evidence, including the aforementioned ones, leads to the novel conception of ALS as a multisystem disease that affects not only primary motor connections but also the connectivity between primary motor regions and supplemental motor and extra-motor regions.

Given that neuropsychological and neuropathological findings may be interpreted as the cognitive and histopathological correlates of disease-related loss of the structural brain integrity in ALS, with a consequent reorganization of cortical networks, we will review current neuropsychological, neuropathological, and neuroimaging knowledge within a framework of cognitive and connectivity changes in ALS, along with some recent hypotheses about pathogenesis.

## 2. Cognitive and Behavioral Changes in ALS

It is now recognized that the ALS-dementia (ALS-D) syndrome is not a random association. It occurs in at least 5% of patients with ALS and includes a set of different subtypes [4, 5]. Clinically, the most common syndrome appears to be very similar to FTD, characterized by personality change, breakdown in social conduct, and impairment of abstraction, planning, set shifting, and organizational skills [2]. Compared to ALS without symptoms of FTD, the prognosis of ALS with comorbid FTD (ALS-FTD) is more unfavorable, and the median survival of patients with ALS-FTD is shorter than that of ALS patients by approximately 1 year [30].

One subgroup of ALS-D patients presents at onset with a predominantly aphasic syndrome, characterized by changes in speech (slowing of speech or dysarthria, anomia, neologisms, echolalia, and semantic paraphasias) [31, 32], although bulbar involvement in the tongue and throat may frequently obscure these language-specific symptoms.

Therefore, patients with ALS can have features of progressive nonfluent aphasia (PNFA), semantic dementia (often atypical), or both. Verbal fluency has been the most frequently investigated executive task in ALS research and has been found to be impaired in the majority of cognitive studies in ALS (e.g., [2, 8, 13]). Furthermore, a number of neuropsychological studies have found deficits among nondemented ALS patients in tasks of confrontation naming, conceptual semantic processing, and syntactic comprehension [33], in conjunction with paraphasias, decreased phrase length, and deficits in phrase construction [5, 34].

In comparison to the findings of impaired executive functioning, memory abilities are less consistently disrupted in ALS [34, 35], with poor performance on memory tasks considered indicative of a failure of encoding information, again implicating a frontal lobe impairment [30].

Behavioral changes are now recognized as another feature of ALS [4]. The term behaviorally impaired (ALSbi) has been recently introduced to describe ALS patients who display frontal behavioral signs but do not meet the full criteria for FTD. Thus, according to current consensus criteria [36], diagnosis of ALSbi requires that the patient meets at least two nonoverlapping supportive diagnostic features from Hodges' criteria [37] for FTD. Although cognitively normal patients with ALS can have profound behavioral abnormalities, cognitive and behavioral impairments can coexist in 25% or more of ALS patients [3].

Disinhibition, irritability, emotional blunting, lack of empathy, and especially apathy have been reported in several cohorts of ALS patients [38–41]. Although the most commonly applied instrument to compare the behavioral changes in several neurodegenerative diseases is the Frontal Systems Behavior (FrSBe) Scale, which is able to assess apathy, disinhibition, and executive dysfunction [42], additional investigations are needed to rule out the potential confounding effects of motor impairment, depression, and recall bias on the evaluation of behavioral modifications by using this in conjunction with measures of mood or other similar scales that account for mood and motor weakness [41].

Furthermore, instrumental markers of cognitive and behavioral impairment in ALS might be useful tools for disease management, promoting the quality of life of both patients and caregivers. In this regard, structural imaging techniques (DTI and voxel-based morphometry, VBM) have allowed to investigate the neuroanatomical correlates of some frontal symptoms, like apathy, the most prominent behavioral feature in ALS [43, 44]. Therefore, in the future, it will be assessed by longitudinal analyses whether DTI and VBM measures may have a predictive value as biomarkers of behavioral impairment in ALS.

## 3. The Neuropathological Basis of Cognitive Impairment in ALS

Together with the advancements of research in neuropsychology, neurobiological studies have inferred a relationship between the density and distribution of pathological abnormalities and cognitive changes in both ALS and FTD. Indeed, by comparing the neuropathologic features of ALS and sporadic FTD, a common and characteristic pathologic finding has emerged in ALS, the ubiquitin-only inclusion body (UBI), at the level of spinal anterior horns, hippocampus, frontotemporal and parietal neocortices [45, 46], and basal ganglia [47, 48], showing higher density and more widespread distribution of inclusions in cognitively impaired ALS patients than in cognitively normal ALS patients [45, 48].

On the basis of these new insights the hypothesis that a common undiscovered proteinopathy underlays both sporadic ALS and FTD was formulated. After TDP was identified as the main disease protein in the majority of FTD cases [49], the ubiquitinated compact and skeinlike inclusions characteristic for ALS were also found to be composed of TDP-43 [49, 50], thereby providing strong evidence that ALS and FTD are part of a clinicopathological continuum of multisystem diseases, the so-called TDP-43 proteinopathies [7]. In fact, immunohistochemical whole-brain analyses of autopsied ALS/FTD cases revealed TDP-43 deposits in multiple brain areas within and also beyond the pyramidal motor system, including the nigrostriatal system, neocortical and allocortical areas, and, to a variable extent, the cerebellum, although there were regional differences in the pathological burden between the various clinical phenotypes [6, 7]. Moreover, the presence or absence of cognitive behavioral dysfunction has been associated with the topographic distribution of cortical TDP-43 inclusions (i.e., predominant involvement of the frontal gyrus in patients with behavioral and/or dysexecutive symptoms, and of the temporal cortex and the angular gyrus in case of language dysfunction) [6, 7, 51].****

Furthermore, cortical involvement with pathological TDP-43 aggregations was usually accompanied by subcortical TDP-43 pathology, in particular in areas directly adjacent to the affected cortex [7]. This finding points toward an involvement of subcortical U-fibers, implicated in connecting multiple cortical areas, suggesting that such subcortical involvement may underlie both cognitive and functional changes in ALS.

Within the ALS/MND-FTD spectrum disorders, multiple genes appear to drive a similar phenotype characterized by neuroglial inclusions immunoreactive to phosphorylated TDP-43. Specifically, mutations in transactivation response DNA-binding protein (TARDBP) gene and in other genes associated with neuronal (NCIs) or glial (GCIs) cytoplasmic inclusions (i.e., fused in sarcoma/translocation in liposarcoma or FUS/TLS, C9ORF72, and progranulin or PGRN) have been identified in several familial or sporadic ALS and ALS/FTD cases [1, 52], and in subsets of FTD [52, 53]. Remarkably, the availability of well-characterized human pathological material in brain banks has yielded the potential to study this disease pathway by creating transgenic animal models, based on different genes but resulting in a common pathology [54–56].

Neuropathological findings from human autopsy studies [51, 57] and experimental models [54–56] of ALS/MND have also suggested that neuronal loss is noncell autonomous and glial cells contribute significantly to neurodegeneration within motor and extra-motor areas. Interestingly, recent evidence suggests that in ALS/MND cytoplasmic protein aggregate inclusions occur also in GCIs in multiple areas. This is especially true for oligodendroglial cells, in some cases showing a significant correlation between the topographic distribution of GCIs and the different clinical subsets of ALS-D [56, 58]. However, taking the neuropathological findings together, it is clear that the idea of a specific frontotemporal dysfunction underlying cognitive impairment in ALS is only partially valid. Instead, this model should be refined in favor of a broader network involving dysfunction in multiple areas, mainly focused on frontotemporal regions that have major connections with posterior areas as well as subcortical and limbic structures.

## 4. Functional Imaging Studies

The whole-brain analysis of functional brain activity has undoubtedly played a crucial role towards a better understanding of the in vivo pathology of ALS over the last two decades.

The earliest single photon emission-computed tomography (SPECT) with 99mTc-hexamethylpropylene, that indirectly evaluated functional brain activity by measuring the regional cerebral uptake of glucose, identified reduced tracer uptake in the frontal lobes of some patients with ALS-D [59, 60]. A number of subsequent SPECT studies have also reported widespread frontotemporal lobe involvement in ALS patients with or without cognitive impairment [10, 11]. Moreover, to explore the relationship between activation and cognitive functions, reduction of regional Cerebral Blood Flow (rCBF) in frontal and temporal areas (anterior and medial orbitofrontal cortex, anterior and medial frontal cortex, and anterior temporal lobes) was correlated to neuropsychological performance, revealing a more marked and widespread pattern of perfusion impairment in patients with ALS-FTD (reduction of rCBF also in the posterior frontal, parietal, and occipital lobes bilaterally) [60, 61] and a significant correlation between memory impairment (abnormal retrieval processes) and frontal hypoperfusion in patients with classical ALS [33]. However, not all previous SPECT studies reported significant correlations between measures of rCBF and neuropsychological data [62], probably because of the different clinical characteristics of the enrolled patients (variability of onset and clinical course of the disease, and of the degree of functional and cognitive impairment) and the different methodologies used.

(18F)2-Fluoro-2-deoxy-D-glucose positron emission tomography (FDG-PET) studies, assessing regional cerebral metabolic rates for glucose (rCMRGlc), also found significantly decreased rCMRGlc in the frontal cortex and superior occipital cortex in classical ALS patients compared to controls, revealing a significant correlation between mild frontal dysfunction and reduced glucose metabolism in the frontal cortex and thalamus [63].

More recently, Flumazenil PET studies assessed the regional flumazenil binding to the benzodiazepine subunit of the Gamma-aminobutyric acid A (GABAA) receptor, as a potential marker for cortical neuronal loss or dysfunction [64, 65]. Reduced [11C]-flumazenil binding in ALS, associated with poorer offline performance on written verbal fluency tasks and Graded Naming Test [66], was reported in the inferior and middle frontal gyri, the superior temporal gyrus, and anterior insula [67], in agreement with earlier neuropathological findings in ALS-aphasia [31, 32]. Further evidence of extra-motor involvement in ALS, using [11C]-flumazenil PET, was also provided by Lloyd et al. [68] who found significant bilateral reductions in the prefrontal cortex, Broca's area, right temporal cortex, the parietal cortex, and right visual association cortex.

In the last two decades, functional activation studies have proven invaluable in exploring disease-related effects in ALS patients on the physiologic activity of different neural systems. The development of new acquisition protocols has allowed the study of the brain, both structurally and functionally, also with the hope of discovering sensitive and specific biomarkers for monitoring the progressive extent of the multisystem degeneration in ALS [69]. However, given that some conditions like hypoxia and hypercapnia might influence brain cognitive and functional modifications [70, 71], their confounding effects should be avoided in the assessment of MRI research projects. Furthermore, it is to take into account that respiratory processes (i.e., natural fluctuations in the depth and rate of breathing and changes in levels of carbon dioxide) can contribute substantially to the measured BOLD signal time series, and removing their effects is an important consideration for fMRI studies of neural function [72, 73].

Remarkably, a widespread frontotemporal lobe involvement has been shown consistently in PET and fMRI studies using both cognitive and motor tasks. For instance, cognitive impairment was examined in a series of studies by Abrahams et al. [12, 13], who compared rCBF during a task of executive function (verbal fluency/word generation) in patients with impaired offline verbal fluency scores (ALSi) and unimpaired offline fluency scores (ALSu). ALSi patients showed reduced activation in the dorsolateral prefrontal cortex (DLPFC), premotor cortex, insular cortex, and thalamus, confirming previous findings [8]. Moreover, Abrahams et al. [74] have also used fMRI to further assess whether word retrieval deficits and underlying cerebral abnormalities are executive in nature, or whether they represent a language dysfunction. They compared ALS patients to matched healthy controls during performance of two tasks: verbal fluency and confrontation naming. The ALS group demonstrated impaired activation in the DLPFC, the anterior cingulate gyrus, and the inferior frontal gyrus (implicated in letter fluency), in the supramarginal gyrus and the temporal lobe auditory association areas (implicated in the phonological store component of working memory and phonological and lexical processing, resp.), and in the occipitotemporal pathway (involved in confrontation naming).

PET and fMRI studies associated with motor tasks have been consistently applied to investigate cortical reorganization of the motor system. In fact, Kew et al. [9], for the first time, demonstrated that ALS patients performing a stereotyped and self-generated PET motor task showed marked activation abnormalities in sensorimotor, parietal association, and anterior cingulate cortices. More recent fMRI studies confirmed these findings about the cortical plasticity in ALS [75, 76]. Moreover, to assess the effects of motor neuron degeneration on both cortical and subcortical areas, Tessitore et al. [77] conducted an fMRI study while a group of ALS patients performed a simple visually paced motor task. In comparison to controls, patients with ALS exhibited reduced activity in the right parietal association cortex, involved in the execution of visually guided movements and strongly connected to the cortical motor area, and heightened activity in the left anterior putamen, also implicated in motor execution. When comparing patients with greater UMN involvement to patients with greater LMN involvement, there were significant differences in the anterior cingulate cortex and right caudate nucleus, with more robust activation of these areas in the group with greater UMN involvement. These results provided further evidence for altered functional responses in brain regions subserving motor behavior in patients with sporadic ALS, reflecting previous morphometric and PET results that had revealed significant impairment of extra-motor regions such as the prefrontal and parietal cortices [46, 67, 68].

The increased striatal activation in patients with ALS was interpreted as a compensatory response to increasing functional demands in the context of affected cortical motor areas, even during the execution of a simple motor task. Therefore, the striatal pattern of activation may indicate the need of the ALS patient group to recruit the basal ganglia system (normally recruited in adaptive control of more complex motor behaviors) as a compensatory circuitry to perform simple motor tasks as well as controls. This finding was consistent with previous PET results concerning an abnormal recruitment of nonprimary motor areas in ALS [9], also interpreted as a pattern of functional adaptation to the corticospinal tract (CST) dysfunction. Thus, it has been suggested that such increased activation during motor tasks may reflect cortical plasticity, as new synapses and pathways are developed to compensate for the selective loss of pyramidal cells in the motor cortex [76] with consequent proliferation of synaptic processes in less affected brain areas. Significantly, patterns of functional adaptation were also detected in ALS by neurophysiological findings, derived from electroencephalography (EEG) and magnetoencephalography (MEG) studies [78, 79], and in case of motor recovery after ischemic stroke [80], in other neurodegenerative disorders [81, 82], and in the aging brain [83].

Further evidence of cortical adaptive changes in the affected brain of ALS patients is derived from fMRI studies that investigated random hand movements against rest [84]. Once again during such a motor task, patients with ALS showed increased cortical activation bilaterally, extending from the sensorimotor cortex posteriorly into the inferior parietal lobule and inferiorly to the superior temporal gyrus. In addition, ALS patients showed reduced activation in the DLPFC extending to anterior and medial frontal cortex [84].

More recently, to assess fMRI longitudinal data on activation changes in different clinical stages of the disease, Mohammadi et al. [85] investigated motor activations in three groups of ALS patients with different degrees of weakness, and a subset of those patients was scanned on multiple occasions. Two distinct stages of neuroplastic changes were identified: first, an increase of the activated area in contralateral sensorimotor cortex, and second, a reduction of signal change and beta weights with increasing weakness. The increase of the activated area was interpreted as a result of decreased intracortical inhibition, which seems to play a determinant role in regulating plasticity in both neurodevelopmental and neurodegenerative disorders [64, 65], and the reduction of movement-related signal change and beta weights as a consequence of loss of upper motor neurons.

A novel focus of neuroimaging research concerns the analysis of functional connectivity of spatially remote brain regions. To this purpose, the whole-brain analysis of functional connectivity by RS-fMRI appears important in developing a better understanding of specific motor or cognitive functions by exploring highly reproducible networks at rest, the so-called resting-state networks (RSNs) [27, 86]. Theoretically, during rest there exist spontaneous coherent fluctuations of the BOLD signal in different brain areas that are functionally connected.

Mohammadi et al. [87] examined, for the first time, the RSNs activity in ALS and demonstrated significant changes in the sensorimotor network (SMN), mainly the premotor area (Brodmann area-BA 6). In addition, in comparison to healthy controls, ALS patients demonstrated a significantly weaker connectivity of the default mode network (DMN) in the ventral anterior cingulate cortex, posterior cingulate cortex, and the left and right inferior parietal cortex, regions that have been linked to higher level executive functions. Indeed, this finding would support previous neuropsychological evidences of a dysexecutive syndrome in ALS patients [2–4, 8, 10, 63].

**Figure 1:**
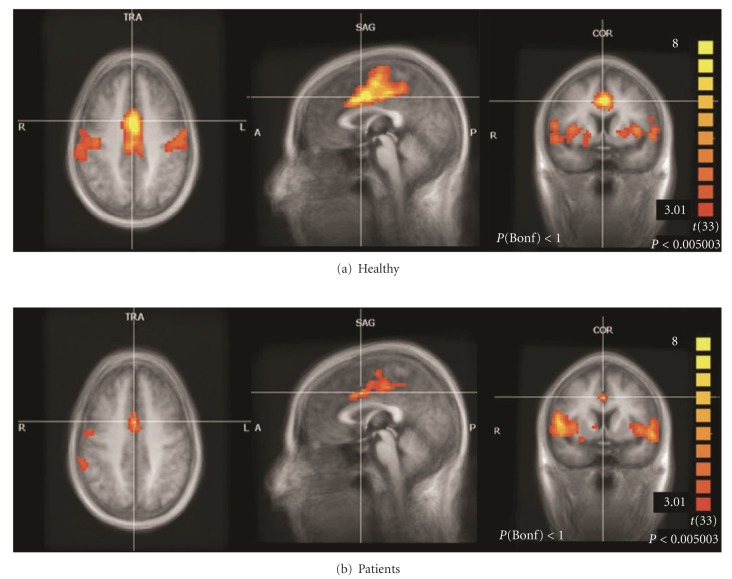
Sensorimotor network (SMN) in the healthy controls (a) and ALS patients (b) groups that Tedeschi et al. [28] examined by RS-fMRI (independent component analysis, ICA). The amount of coherent RS-fMRI fluctuations within this network appeared strongly reduced in the ALS population.

Later, Tedeschi et al. [28] designed an RS-fMRI study in ALS not only to assess functional RSNs but also to examine the possible interaction between neurodegeneration and aging, which has been reported to induce physiological age-related modulation effects on fMRI signal fluctuations especially in the DMN [88, 89]. The amount of coherent RS-fMRI fluctuations within the SMN network appeared strongly and significantly reduced in the ALS population especially in primary motor cortex (PMC) regions (Figure 1), in agreement with those reported by Mohammadi et al. [87]. Furthermore, the frontoparietal network (FPN), which includes the main RSNs in the cognitive domain, presented a selective suppression of signal fluctuations in ALS patients in two clusters of the right FPN (the superior frontal gyrus and the supramarginal gyrus). These effects in a cognitive executive network like the right FPN are consistent with the frontal executive dysfunction that has been largely described in ALS [2–4].

Remarkably, in the ALS patient group there was a statistically significant interaction between neurodegeneration and aging (disease-by-age effect) in the DMN, specifically, in the posterior cingulate cortex (PCC). In fact, this effect resulted capable of inverting the trend of negative correlation between functional connectivity and age observed in the sex- and age-matched control group. This finding is in line with recent evidence that neurodegenerative dementias may be associated with increased functional connectivity within unaffected (or affected at later stages) networks with less evident functional decline [90, 91]. Particularly, posterior cortical functions have been shown to survive or even thrive in patients with FTD [92, 93] in contrast to Alzheimer's disease that, like normal aging, damages the posterior part of the DMN [94].

Interestingly, the positive modulation on the spontaneous functional connectivity of the posterior part of DMN described by Tedeschi et al. [28] in an ALS population appears to be similar to the RS-fMRI trend observed in the behavioral variant of FTD [94] and may be interpreted as the functional expression of a possible compensatory mechanism of the default system to the combined effect of degeneration and aging. However, a recent study using animal and cellular models of ALS pathophysiology [95] has linked neurodegeneration and aging to specific strategies of neuroprotection by which the cell damage is contrasted with adaptive mechanisms against the physiological stress implied by aging.

## 5. Structural Neuroimaging

Morphometric studies by volumetric MRI were originally used in ALS for the in vivo investigation of region-specific volume reductions and have enabled the detection of subtle yet significant cortical and subcortical changes in the frontal and temporal lobes [96, 97].

In recent years, the development of advanced automated imaging analysis, based upon construction of statistical parametric maps, allowed detailed anatomic studies of brain morphometry. Particularly, voxel-based morphometry (VBM) allows a fully automated whole-brain measurement of regional brain atrophy by voxelwise comparison of gray matter (GM) and white matter (WM) volumes between groups of subjects [98]. The most consistent finding of VBM studies in ALS involves GM atrophy in several regions of the frontal (i.e., the anterior cingulate, middle and inferior frontal gyrus, BA 8, 9, and 10) and temporal lobes (i.e., temporal poles, superior temporal gyrus, temporal isthmus) [14–17, 28, 99].

Among the authors who investigated GM volumetric changes in ALS, Mezzapesa et al. [15] and Grossman et al. [16] reported significant correlations between measures of cognitive function and cortical atrophy in classical ALS patients.

Mezzapesa et al. [15] detected a gray matter volume decrease in several frontal and temporal areas bilaterally in patients with ALS, whose performances on Symbol Digit Modalities Test were significantly worse compared with controls. Therefore, the presence of mild whole-brain volume loss and regional frontotemporal atrophy seemed to be related to the cognitive impairment in patients with ALS.

Grossman et al. [16] showed atrophy in several regions including the frontal, temporal, limbic, and occipital lobes. From a neuropsychological point of view, patients showed significant difficulty on measures requiring action knowledge compared to object knowledge, and performances to this kind of tasks were highly correlated with cortical atrophy in motor regions. Interestingly, scores on tests of both action and object knowledge were correlated with decreased GM volume in inferior frontal cortex and DLPFC, known to be involved in components of semantic memory. Therefore, deficiency in semantic access in patients with ALS partially reflects the degeneration of motor system mediation of tasks requiring knowledge of action features, while also reflecting degeneration of prefrontal regions responsible for both action and object knowledge.

To identify a marker of upper motor neuron degeneration, a surface-based cortical morphology technique has also been applied in ALS measuring cortical thickness, surface, and volume. Cortical morphology analyses revealed specific thinning in the precentral gyrus (preCG) [21, 100, 101] correlated with CST damage evaluated by DTI in combined analyses [21, 100]. A significant direct association was not found between measures of cortical thickness and cognitive impairment, although relative thinning in temporal regions was associated with a rapidly progressive disease course [101].

DTI studies of ALS have developed along two main directions: (i) a voxel-by-voxel evaluation of whole-brain WM and (ii) measurements of specific tracts by positioning regions of interest (ROIs). The first type of analysis involves the coregistration of each person's scan to a common template and can be performed without an a priori hypothesis. With this method, anisotropy maps are coregistered into a standard space, allowing comparisons of anisotropy value between groups. Moreover, this approach based on whole-brain DTI analysis may result in higher accuracy in detecting widespread microstructural disease-related WM changes rather than by using an ROI-based method.

Recent whole-brain DTI analyses reported regions of WM damage in ALS via voxel-based [17–19, 23, 102, 103], tract-based spatial statistics (TBSS) [18, 20, 22, 24, 104], and High Angular Resolution Diffusion Imaging (HARDI) [25] approaches. Most of these studies found changes of fractional anisotropy (FA) and mean diffusivity (MD) not only in the CSTs but also in the corpus callosum [18, 20, 23, 24, 102] and the frontal and temporal lobes [17, 18, 23, 29].

**Figure 2:**
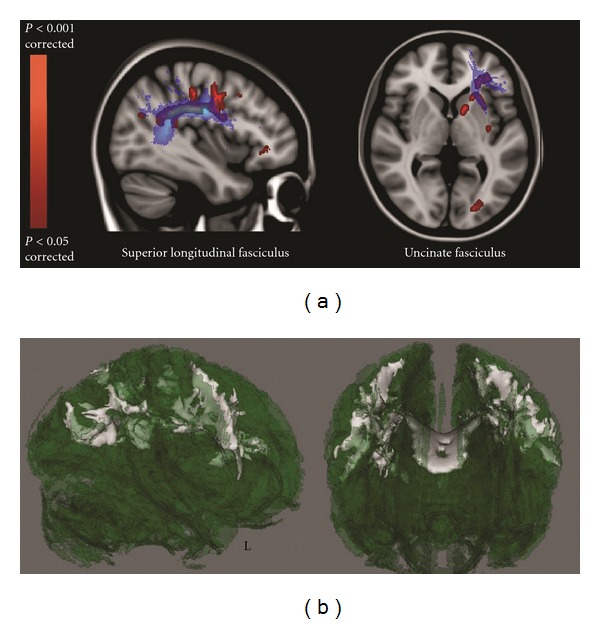
Regional FA reductions in ALS patients compared with healthy controls in frontal (associative) tracts (TBSS DTI analysis performed by Cirillo et al. [24]). In (a), blue shows the superior longitudinal and the uncinate fasciculi (derived from the Johns Hopkins University White-Matter Tractography atlas [106, 107]), whilst red shows significant FA decrease in ALS patients (*P* < 0.05, corrected). (b) illustrates 3D renderings of the FA skeleton (green), where white shows regional FA reductions in patients. Remarkably, these diffusivity changes resemble those which have been described in patients with the behavioral variant of frontotemporal dementia.

Recently, new insights in the assessment of corticomotor connectivity changes in ALS were obtained by acquiring HARDI scans along with high-resolution structural images (sMRI) [25]. A significant reduction in mean FA within a number of intra- and interhemispheric WM connections associated with the preCG and postcentral (postCG) gyri was found in ALS participants compared to controls, in agreement with other DTI analyses (i.e., FA decrease in anterior cingulate, superior longitudinal, inferior longitudinal, inferior occipitofrontal, and uncinate fasciculi) (Figure 2) [24, 25]. Once again this DTI pattern of predominantly frontal WM injury clearly reflects the frontal executive dysfunction that has been extensively described in several cohorts of patients with ALS [2, 3] and is consistent with similar diffusivity changes described in patients with the behavioral variant of FTD [105].

By combining DTI and graph analytical network approaches (examination of the organization of widespread functional brain networks or connectome), Verstraete et al. [29] found a significantly impaired structural network overlapping bilateral primary motor regions (precentral gyrus and paracentral lobule, BA 4), bilateral supplementary motor regions (caudal middle frontal gyrus, BA 6), parts of the left basal ganglia (pallidum), and right posterior cingulate and precuneus in ALS. Therefore, the neurodegeneration process seems to affect not only the primary motor connections but also the connectivity between primary motor regions and supplemental motor areas. The authors hypothesize that the disease starts in the precentral gyrus and progresses along the structural connections of the primary motor regions towards secondary motor regions, as suggested by both DTI [20, 24] and graph analytical network evidence. Alternatively, brain plasticity might be potentially attributed to the reduced motor connectivity. This takes into account that the connectivity changes reported in DMN in ALS patients [28, 87] were in agreement with the findings of impaired structural connectivity of the motor network to the precuneus and PCC, key regions of the DMN.

**Figure 3:**
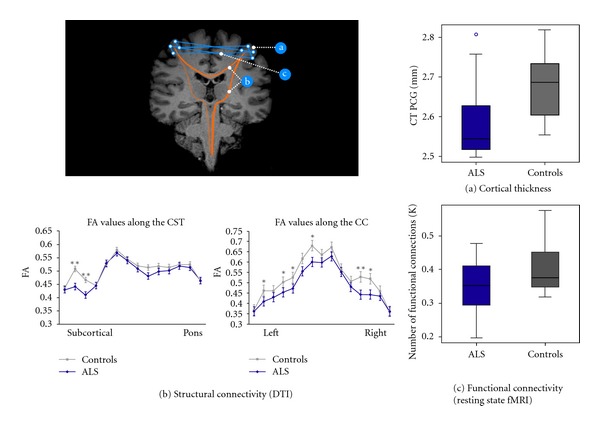
(a) Cortical thickness (CT) in patients with ALS versus controls in the preCG in mm (*P* = 0.04), corrected for age and whole brain CT. (b) Fractional anisotropy (FA) values along the CST and the corpus callosum, evaluated by DTI analysis, in patients with ALS and controls (**P < 0.01; *P < 0.05). (c) Number of functional connections in patients with ALS versus controls, corrected for age (threshold 0.40) (*P* = 0.14): this result was indicative of a relative sparing of functional connectivity in patients (derived from Verstraete et al. [21]).

**Figure 4:**
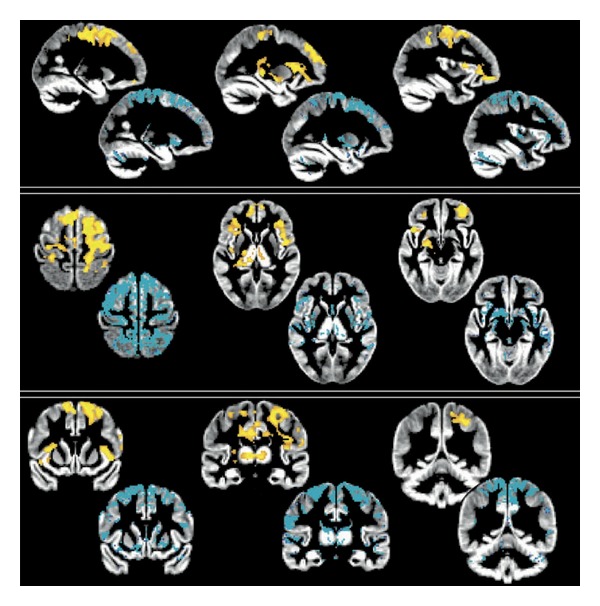
Increase of functional connectivity and lower structural connectivity in a population of ALS patients studied by Douaud et al. [22]. The spatial distribution of the significant increase of functional connectivity in patients (red-yellow scale: *P* < 0.05, corrected) corresponded to the areas where the patients had lower structural connectivity, evaluated by using tract-based spatial statistics and probabilistic tractography, in comparison to healthy controls (in blue, thresholded at 10 streamlines of difference on average) (derived from Douaud et al. [22]). By permission of Oxford University Press.

To investigate the functional correlates of the structural changes, combined MRI studies have been recently performed in ALS. A multiparametric analysis by Verstraete et al. [21], based on a network perspective, by combining cortical thickness, DTI and RS-fMRI techniques, demonstrated a decline of structural integrity (i.e., significant reduction of cortical thickness in the preCG and in microstructural organization of rostral CST) with preserved functional organization of the motor network in ALS (Figure 3). Moreover, the local connectedness was found to be related with disease progression. Accordingly with these results, another MRI study of connectivity in ALS by Douaud et al. [22] demonstrated an increased functional connectivity directly associated with an impaired ALS-specific grey matter network (predefined by the consistent regions of WM damage), spanning sensorimotor, premotor, prefrontal, and thalamic regions (Figure 4). Patients with a slower rate of disease progression (not only longer disease duration) presented connectivity values more comparable to those of healthy controls. Therefore, these findings prompted speculation as to whether connectivity changes might have a more active role in pathogenesis. In fact, one hypothesis is that increased functional connectivity arises as a result of loss of central nervous system interneurons influence, reflected in the hitherto unexplained variable compartmentalization of pathology within upper and lower motor neuron populations [108]. This interneuronopathy may cause a generalized hyperexcitability in the motor cortex, as also shown by several electrophysiological findings (i.e., derived from transcranial magnetic stimulation or TMS, and event-related potentials or ERP studies) [109–111], and appears also corroborated by histopathological [41] and flumazenil PET [67, 68] evidence.

Studies of lower motoneurons in the animal model of the disease have given important clues to the downstream mechanisms of cell death in the spinal cord, where the earliest damage appears to occur in the interneurons in lamina VII known as Renshaw cells [112]. It was then hypothesized that dysfunction or loss of Renshaw cells may have important consequences for spinal connectivity and motor control. Speculatively therefore, an excitotoxic pathway common to upper and lower motor neurons populations might result from an unopposed glutamatergic activity [108]. However, abnormal presymptomatic development of lower motor neuron connectivity has not been seemed a prerequisite for subsequent neuromuscular pathology in a mouse model of severe spinal muscular atrophy (SMA) [113].

Finally, an era of multimodal MRI studies, combining several advanced techniques, along with neuropsychological, genetic, and histopathological information, might lead to a comprehensive assessment of neurodegeneration in ALS, including disease mechanisms and monitoring of disease progression and therapeutics. Remarkably, with the model of Alzheimer's Disease Neuroimaging Initiative (ADNI) [114] in mind, Oxford University (UK) hosted international scientists at the first Neuroimaging Symposium in ALS (NISALS; November 2010), which led to the development of consensus guidelines on image acquisition and analysis, with the aim of retrospective data sharing to further explore the feasibility of MRI as a surrogate marker in future therapeutic trials for ALS [69].

## 6. Concluding Remarks

The involvement of frontotemporal areas in ALS and the existence of overlap syndromes with dementia types have been recognized for decades. Functional imaging studies have confirmed that functional changes beyond the primary motor network are a common feature in ALS patients and may reflect an attempt of the ALS brain to compensate for the effect of motor neurodegeneration by neural plasticity within unaffected or less affected structures subserving cognitive domains. Alternatively, the abnormal functional connectivity may arise as a result of loss of interneurons inhibitory influence, with widespread and variable effects on upper and lower motor neuron populations.

We believe that future studies based on neuropsychology, advanced imaging, molecular pathology, and genetics will further enhance our understanding of the relationship between motor system dysfunction and cognition and provide valuable information on the physiopathological mechanisms underlying the complex interaction between the multiple affected systems in ALS.

## References

[1] Renton AE, Majounie E, Waite A (2011). A hexanucleotide repeat expansion in C9ORF72 is the cause of chromosome 9p21-linked ALSFTD. *Neuron*.

[2] Abrahams S, Goldstein LH, Suckling J (2005). Frontotemporal white matter changes in amyotrophic lateral sclerosis. *Journal of Neurology*.

[3] Murphy J, Henry R, Lomen-Hoerth C (2007). Establishing subtypes of the continuum of frontal lobe impairment in amyotrophic lateral sclerosis. *Archives of Neurology*.

[4] Phukan J, Pender NP, Hardiman O (2007). Cognitive impairment in amyotrophic lateral sclerosis. *The Lancet Neurology*.

[5] Ringholz GM, Appel SH, Bradshaw M, Cooke NA, Mosnik DM, Schulz PE (2005). Prevalence and patterns of cognitive impairment in sporadic ALS. *Neurology*.

[6] Geser F, Brandmeir NJ, Kwong LK (2008). Evidence of multisystem disorder in whole-brain map of pathological TDP-43 in amyotrophic lateral sclerosis. *Archives of Neurology*.

[7] Geser F, Martinez-Lage M, Robinson J (2009). Clinical and pathological continuum of multisystem TDP-43 proteinopathies. *Archives of Neurology*.

[8] Kew JJM, Goldstein LH, Leigh PN (1993). The relationship between abnormalities of cognitive function and cerebral activation in amyotrophic lateral sclerosis: a neuropsychological and positron emission tomography study. *Brain*.

[9] Kew JJM, Leigh PN, Playford ED (1993). Cortical function in amyotrophic lateral sclerosis. A positron emission tomography study. *Brain*.

[10] Vercelletto M, Ronin M, Huvet M, Magne C, Feve JR (1999). Frontal type dementia preceding amyotrophic lateral sclerosis: a neuropsychological and SPECT study of five clinical cases. *European Journal of Neurology*.

[11] Vercelletto M, Belliard S, Wiertlewski S (2003). Neuropsychological and scintigraphic aspects of frontotemporal dementia preceding amyotrophic lateral sclerosis. *Revue Neurologique*.

[12] Abrahams S, Goldstein LH, Kew JJM (1996). Frontal lobe dysfunction in amyotrophic lateral sclerosis: a PET study. *Brain*.

[13] Abrahams S, Leigh PN, Kew JJM, Goldstein LH, Lloyd CML, Brooks DJ (1995). A positron emission tomography study of frontal lobe function (verbal fluency) in amyotrophic lateral sclerosis. *Journal of the Neurological Sciences*.

[14] Chang JL, Lomen-Hoerth C, Murphy J (2005). A voxel-based morphometry study of patterns of brain atrophy in ALS and ALS/FTLD. *Neurology*.

[15] Mezzapesa DM, Ceccarelli A, Dicuonzo F (2007). Whole-brain and regional brain atrophy in amyotrophic lateral sclerosis. *American Journal of Neuroradiology*.

[16] Grossman M, Anderson C, Khan A, Avants B, Elman L, McCluskey L (2008). Impaired action knowledge in amyotrophic lateral sclerosis. *Neurology*.

[17] Agosta F, Pagani E, Rocca MA (2007). Voxel-based morphometry study of brain volumetry and diffusivity in amyotrophic lateral sclerosis patients with mild disability. *Human Brain Mapping*.

[18] Sage CA, van Hecke W, Peeters R (2009). Quantitative diffusion tensor imaging in amyotrophic lateral sclerosis: revisited. *Human Brain Mapping*.

[19] Agosta F, Pagani E, Petrolini M (2010). Assessment of white matter tract damage in patients with amyotrophic lateral sclerosis: a diffusion tensor MR imaging tractography study. *American Journal of Neuroradiology*.

[20] Filippini N, Douaud G, MacKay CE, Knight S, Talbot K, Turner MR (2010). Corpus callosum involvement is a consistent feature of amyotrophic lateral sclerosis. *Neurology*.

[21] Verstraete E, van den Heuvel MP, Veldink JH (2010). Motor network degeneration in amyotrophic lateral sclerosis: a structural and functional connectivity study. *PLoS ONE*.

[22] Douaud G, Filippini N, Knight S, Talbot K, Turner MR (2011). Integration of structural and functional magnetic resonance imaging in amyotrophic lateral sclerosis. *Brain*.

[23] Canu E, Agosta F, Riva N (2011). The topography of brain microstructural damage in amyotrophic lateral sclerosis assessed using diffusion tensor MR imaging. *American Journal of Neuroradiology*.

[24] Cirillo M, Esposito F, Tedeschi G Widespread microstructural white matter involvement in amyotrophic lateral sclerosis: a whole-brain DTI study.

[25] Rose S, Pannek K, Bell C (2012). Direct evidence of intra- and interhemispheric corticomotor network degeneration in amyotrophic lateral sclerosis: an automated MRI structural connectivity study. *NeuroImage*.

[26] Greicius MD, Krasnow B, Reiss AL, Menon V (2003). Functional connectivity in the resting brain: a network analysis of the default mode hypothesis. *Proceedings of the National Academy of Sciences of the United States of America*.

[27] Mantini D, Perrucci MG, Del Gratta C, Romani GL, Corbetta M (2007). Electrophysiological signatures of resting state networks in the human brain. *Proceedings of the National Academy of Sciences of the United States of America*.

[28] Tedeschi G, Trojsi F, Tessitore A (2012). Interaction between aging and neurodegeneration in amyotrophic lateral sclerosis. *Neurobiology of Aging*.

[29] Verstraete E, Veldink JH, Mandl RCW, van den Berg LH, van den Heuvel MP (2011). Impaired structural motor connectome in amyotrophic lateral sclerosis. *PLoS ONE*.

[30] Josephs KA, Knopman DS, Whitwell JL (2005). Survival in two variants of tau-negative frontotemporal lobar degeneration: FTLD-U vs FTLD-MND. *Neurology*.

[31] Bak TH, Hodges JR (2001). Motor neurone disease, dementia and aphasia: coincidence, co-occurrence or continuum?. *Journal of Neurology*.

[32] Caselli RJ, Windebank AJ, Petersen RC (1993). Rapidly progressive aphasic dementia and motor neuron disease. *Annals of Neurology*.

[33] Mantovan MC, Baggio L, Dalla Barba G (2003). Memory deficits and retrieval processes in ALS. *European Journal of Neurology*.

[34] Gallassi R, Montagna P, Morreale A (1989). Neuropsychological, electroencephalogram and brain computed tomography findings in motor neuron disease. *European Neurology*.

[35] Hanagasi HA, Gurvit IH, Ermutlu N (2002). Cognitive impairment in amyotrophic lateral sclerosis: evidence from neuropsychological investigation and event-related potentials. *Cognitive Brain Research*.

[36] Strong MJ (2009). Consensus criteria for the diagnosis of frontotemporal cognitive and behavioural syndromes in amyotrophic lateral sclerosis. *Amyotrophic Lateral Sclerosis*.

[37] Hodges JR, Miller B (2001). The classification, genetics and neuropathology of frontotemporal dementia. Introduction to the special topic papers: part I. *Neurocase*.

[38] Grossman AB, Woolley-Levine S, Bradley WG, Miller RG (2007). Detecting neurobehavioral changes in amyotrophic lateral sclerosis. *Amyotrophic Lateral Sclerosis*.

[39] Gibbons ZC, Richardson A, Neary D, Snowden JS (2008). Behaviour in amyotrophic lateral sclerosis. *Amyotrophic Lateral Sclerosis*.

[40] Lillo P, Mioshi E, Zoing MC, Kiernan MC, Hodges JR (2011). How common are behavioural changes in amyotrophic lateral sclerosis?. *Amyotrophic Lateral Sclerosis*.

[41] Witgert M, Salamone AR, Strutt AM (2010). Frontal-lobe mediated behavioral dysfunction in amyotrophic lateral sclerosis. *European Journal of Neurology*.

[42] Grace J, Stout JC, Malloy PF (1999). Assessing frontal lobe behavioral syndromes with the frontal lobe personality scale. *Assessment*.

[43] Woolley SC, Zhang Y, Schuff N, Weiner MW, Katz JS (2011). Neuroanatomical correlates of apathy in ALS using 4 Tesla diffusion tensor MRI. *Amyotrophic Lateral Sclerosis*.

[44] Tsujimoto M, Senda J, Ishihara T (2011). Behavioral changes in early ALS correlate with voxel-based morphometry and diffusion tensor imaging. *Journal of the Neurological Sciences*.

[45] Wilson CM, Grace GM, Munoz DG, He BP, Strong MJ (2001). Cognitive impairment in sporadic ALS: a pathologic continuum underlying a multisystem disorder. *Neurology*.

[46] Maekawa S, Al-Sarraj S, Kibble M (2004). Cortical selective vulnerability in motor neuron disease: a morphometric study. *Brain*.

[47] Al-Sarraj S, Maekawa S, Kibble M, Everall I, Leigh N (2002). Ubiquitin-only intraneuronal inclusion in the substantia nigra is a characteristic feature of motor neurone disease with dementia. *Neuropathology and Applied Neurobiology*.

[48] Kawashima T, Doh-ura K, Kikuchi H, Iwaki T (2001). Cognitive dysfunction in patients with amyotrophic lateral sclerosis is associated with spherical or crescent-shaped ubiquitinated intraneuronal inclusions in the parahippocampal gyrus and amygdala, but not in the neostriatum. *Acta Neuropathologica*.

[49] Neumann M, Sampathu DM, Kwong LK (2006). Ubiquitinated TDP-43 in frontotemporal lobar degeneration and amyotrophic lateral sclerosis. *Science*.

[50] Mackenzie IRA, Bigio EH, Ince PG (2007). Pathological TDP-43 distinguishes sporadic amyotrophic lateral sclerosis from amyotrophic lateral sclerosis with SOD1 mutations. *Annals of Neurology*.

[51] Brettschneider J, Libon DJ, Toledo JB (2012). Microglial activation and TDP-43 pathology correlate with executive dysfunction in amyotrophic lateral sclerosis. *Acta Neuropathologica*.

[52] Mackenzie IRA, Rademakers R, Neumann M (2010). TDP-43 and FUS in amyotrophic lateral sclerosis and frontotemporal dementia. *The Lancet Neurology*.

[53] Baker M, Mackenzie IR, Pickering-Brown SM (2006). Mutations in progranulin cause tau-negative frontotemporal dementia linked to chromosome 17. *Nature*.

[54] Blackburn D, Sargsyan S, Monk PN, Shaw PJ (2009). Astrocyte function and role in motor neuron disease: a future therapeutic target?. *Glia*.

[55] Ilieva H, Polymenidou M, Cleveland DW (2009). Non-cell autonomous toxicity in neurodegenerative disorders: ALS and beyond. *The Journal of Cell Biology*.

[56] Zhang H, Tan CF, Mori F (2008). TDP-43-immunoreactive neuronal and glial inclusions in the neostriatum in amyotrophic lateral sclerosis with and without dementia. *Acta Neuropathologica*.

[57] Troakes C, Maekawa S, Wijesekera L An MND/ALS phenotype associated with C9orf72 repeat expansion: abundant p62-positive, TDP-43-negative inclusions in cerebral cortex, hippocampus and cerebellum but without associated cognitive decline.

[58] Ince PG, Highley JR, Kirby J (2011). Molecular pathology and genetic advances in amyotrophic lateral sclerosis: an emerging molecular pathway and the significance of glial pathology. *Acta Neuropathologica*.

[59] Neary D, Snowden JS, Mann DMA, Northern B, Goulding PJ, Macdermott N (1990). Frontal lobe dementia and motor neuron disease. *Journal of Neurology Neurosurgery and Psychiatry*.

[60] Talbot PR, Goulding PJ, Lloyd JJ, Snowden JS, Neary D, Testa HJ (1995). Inter-relation between “classic” motor neuron disease and frontotemporal dementia: neuropsychological and single photon emission computed tomography study. *Journal of Neurology Neurosurgery and Psychiatry*.

[61] Ishikawa T, Morita M, Nakano I (2007). Constant blood flow reduction in premotor frontal lobe regions in ALS with dementia—a SPECT study with 3D-SSP. *Acta Neurologica Scandinavica*.

[62] Rusina R, Ridzoň P, Kulišt’Ák P (2010). Relationship between ALS and the degree of cognitive impairment, markers of neurodegeneration and predictors for poor outcome. A prospective study. *European Journal of Neurology*.

[63] Ludolph AC, Langen KJ, Regard M (1992). Frontal lobe function in amyotrophic lateral sclerosis: a neuropsychologic and positron emission tomography study. *Acta Neurologica Scandinavica*.

[64] Allain AE, Le Corronc H, Delpy A (2011). Maturation of the GABAergic transmission in normal and pathologic motoneurons. *Neural Plasticity*.

[65] Baroncelli L, Braschi C, Spolidoro M, Begenisic T, Maffei L, Sale A (2011). Brain plasticity and disease: a matter of inhibition. *Neural Plasticity*.

[66] McKenna P, Warrington EK (1980). Testing for nominal dysphasia. *Journal of Neurology Neurosurgery and Psychiatry*.

[67] Wicks P, Turner MR, Abrahams S (2008). Neuronal loss associated with cognitive performance in amyotrophic lateral sclerosis: an (11C)-flumazenil PET study. *Amyotrophic Lateral Sclerosis*.

[68] Lloyd CM, Richardson MP, Brooks DJ, Al-Chalabi A, Leigh PN (2000). Extramotor involvement in ALS: PET studies with the GABA(A) ligand [11C]flumazenil. *Brain*.

[69] Turner MR, Grosskreutz J, Kassubek J (2011). Towards a neuroimaging biomarker for amyotrophic lateral sclerosis. *The Lancet Neurology*.

[70] Yan X, Zhang J, Shi J, Gong Q, Weng X (2010). Cerebral and functional adaptation with chronic hypoxia exposure: a multi-modal MRI study. *Brain Research*.

[71] Xu F, Uh J, Brier MR (2011). The influence of carbon dioxide on brain activity and metabolism in conscious humans. *Journal of Cerebral Blood Flow and Metabolism*.

[72] Chang C, Glover GH (2009). Relationship between respiration, end-tidal CO2, and BOLD signals in resting-state fMRI. *NeuroImage*.

[73] Birn RM, Diamond JB, Smith MA, Bandettini PA (2006). Separating respiratory-variation-related fluctuations from neuronal-activity-related fluctuations in fMRI. *NeuroImage*.

[74] Abrahams S, Goldstein LH, Simmons A (2004). Word retrieval in amyotrophic lateral sclerosis: a functional magnetic resonance imaging study. *Brain*.

[75] Konrad C, Henningsen H, Bremer J (2002). Pattern of cortical reorganization in amyotrophic lateral sclerosis: a functional magnetic resonance imaging study. *Experimental Brain Research*.

[76] Schoenfeld MA, Tempelmann C, Gaul C (2005). Functional motor compensation in amyotrophic lateral sclerosis. *Journal of Neurology*.

[77] Tessitore A, Esposito F, Monsurrò MR (2006). Subcortical motor plasticity in patients with sporadic ALS: an fMRI study. *Brain Research Bulletin*.

[78] Inuggi A, Riva N, González-Rosa JJ (2011). Compensatory movement-related recruitment in amyotrophic lateral sclerosis patients with dominant upper motor neuron signs: an EEG source analysis study. *Brain Research*.

[79] Teismann IK, Warnecke T, Suntrup S (2011). Cortical processing of swallowing in ALS patients with progressive dysphagia—a magnetoencephalographic study. *PLoS ONE*.

[80] Hosp JA, Luft AR (2011). Cortical plasticity during motor learning and recovery after ischemic stroke. *Neural Plasticity*.

[81] Bondi MW, Houston WS, Eyler LT, Brown GG (2005). fMRI evidence of compensatory mechanisms in older adults at genetic risk for Alzheimer disease. *Neurology*.

[82] Mattay VS, Tessitore A, Callicott JH (2002). Dopaminergic modulation of cortical function in patients with Parkinson’s disease. *Annals of Neurology*.

[83] Ward NS, Frackowiak RSJ (2003). Age-related changes in the neural correlates of motor performance. *Brain*.

[84] Stanton BR, Williams VC, Leigh PN (2007). Altered cortical activation during a motor task in ALS: evidence for involvement of central pathways. *Journal of Neurology*.

[85] Mohammadi B, Kollewe K, Samii A, Dengler R, Münte TF (2011). Functional neuroimaging at different disease stages reveals distinct phases of neuroplastic changes in amyotrophic lateral sclerosis. *Human Brain Mapping*.

[86] Damoiseaux JS, Rombouts SARB, Barkhof F (2006). Consistent resting-state networks across healthy subjects. *Proceedings of the National Academy of Sciences of the United States of America*.

[87] Mohammadi B, Kollewe K, Samii A, Krampfl K, Dengler R, Münte TF (2009). Changes of resting state brain networks in amyotrophic lateral sclerosis. *Experimental Neurology*.

[88] Esposito F, Aragri A, Pesaresi I (2008). Independent component model of the default-mode brain function: combining individual-level and population-level analyses in resting-state fMRI. *Magnetic Resonance Imaging*.

[89] Sambataro F, Murty VP, Callicott JH (2010). Age-related alterations in default mode network: impact on working memory performance. *Neurobiology of Aging*.

[90] Seeley WW, Crawford RK, Zhou J, Miller BL, Greicius MD (2009). Neurodegenerative diseases target large-scale human brain networks. *Neuron*.

[91] Wang L, Zang Y, He Y (2006). Changes in hippocampal connectivity in the early stages of Alzheimer’s disease: evidence from resting state fMRI. *NeuroImage*.

[92] Miller BL, Cummings J, Mishkin F (1998). Emergence of artistic talent in frontotemporal dementia. *Neurology*.

[93] Seeley WW, Matthews BR, Crawford RK (2008). Unravelling Boléro: progressive aphasia, transmodal creativity and the right posterior neocortex. *Brain*.

[94] Zhou J, Greicius MD, Gennatas ED (2010). Divergent network connectivity changes in behavioural variant frontotemporal dementia and Alzheimer’s disease. *Brain*.

[95] Madeo F, Eisenberg T, Kroemer G (2009). Autophagy for the avoidance of neurodegeneration. *Genes and Development*.

[96] Kiernan JA, Hudson AJ (1994). Frontal lobe atrophy in motor neuron diseases. *Brain*.

[97] Kato S, Hayahi H, Yagishita A (1993). Involvement of the frontotemporal lobe and limbic system in amyotrophic lateral sclerosis: as assessed by serial computed tomography and magnetic resonance imaging. *Journal of the Neurological Sciences*.

[98] Ashburner J, Friston KJ (2000). Voxel-based morphometry—the methods. *NeuroImage*.

[99] Thivard L, Pradat PF, Lehéricy S (2007). Diffusion tensor imaging and voxel based morphometry study in amyotrophic lateral sclerosis: relationships with motor disability. *Journal of Neurology, Neurosurgery and Psychiatry*.

[100] Roccatagliata L, Bonzano L, Mancardi G, Canepa C, Caponnetto C (2009). Detection of motor cortex thinning and corticospinal tract involvement by quantitative MRI in amyotrophic lateral sclerosis. *Amyotrophic Lateral Sclerosis*.

[101] Verstraete E, Veldink JH, Hendrikse J, Schelhaas HJ, van den Heuvel MP, van den Berg LH (2012). Structural MRI reveals cortical thinning in amyotrophic lateral sclerosis. *Journal of Neurology, Neurosurgery and Psychiatry*.

[102] Sach M, Winkler G, Glauche V (2004). Diffusion tensor MRI of early upper motor neuron involvement in amyotrophic lateral sclerosis. *Brain*.

[103] Sage CA, Peeters RR, Görner A, Robberecht W, Sunaert S (2007). Quantitative diffusion tensor imaging in amyotrophic lateral sclerosis. *NeuroImage*.

[104] Smith SM, Jenkinson M, Johansen-Berg H (2006). Tract-based spatial statistics: voxelwise analysis of multi-subject diffusion data. *NeuroImage*.

[105] Whitwell JL, Avula R, Senjem ML (2010). Gray and white matter water diffusion in the syndromic variants of frontotemporal dementia. *Neurology*.

[106] Hua K, Zhang J, Wakana S (2008). Tract probability maps in stereotaxic spaces: analyses of white matter anatomy and tract-specific quantification. *NeuroImage*.

[107] Wakana S, Caprihan A, Panzenboeck MM (2007). Reproducibility of quantitative tractography methods applied to cerebral white matter. *NeuroImage*.

[108] Turner MR, Kiernan MC (2012). Does interneuronal dysfunction contribute to neurodegeneration in amyotrophic lateral sclerosis?. *Amyotrophic Lateral Sclerosis*.

[109] Yokota T, Yoshino A, Inaba A, Saito Y (1996). Double cortical stimulation in amyotrophic lateral sclerosis. *Journal of Neurology Neurosurgery and Psychiatry*.

[110] Khedr EM, Ahmed MA, Hamdy A, Shawky OA (2011). Cortical excitability of amyotrophic lateral sclerosis: transcranial magnetic stimulation study. *Neurophysiologie Clinique*.

[111] Riva N, Falini A, Inuggi A Cortical activation to voluntary movement in amyotrophic lateral sclerosis is related to corticospinal damage: electrophysiologicalevidence.

[112] Mazzocchio R, Rossi A (2010). Role of renshaw cells in amyotrophic lateral sclerosis. *Muscle and Nerve*.

[113] Murray LM, Lee S, Baumer D, Parson SH, Talbot K, Gillingwater TH (2009). Pre-symptomatic development of lower motor neuron connectivity in a mouse model of severe spinal muscular atrophy. *Human Molecular Genetics*.

[114] Mueller SG, Weiner MW, Thal LJ (2005). The Alzheimer’s disease neuroimaging initiative. *Neuroimaging Clinics of North America*.

